# Efficiency and equity of community-based falls prevention pathways: a model-based health economic evaluation

**DOI:** 10.1093/ageing/afaf212

**Published:** 2025-08-03

**Authors:** Joseph Kwon, Hazel Squires, Tracey Young

**Affiliations:** Nuffield Department of Primary Care Health Sciences, University of Oxford, Radcliffe Primary Care Building, Woodstock Road, Oxford OX2 6GG, UK; Sheffield Centre for Health and Related Research, The University of Sheffield, Sheffield, UK; Sheffield Centre for Health and Related Research, The University of Sheffield, Sheffield, UK

**Keywords:** falls prevention, economic model, NICE falls prevention guideline, equity, older people

## Abstract

**Background:**

Three pathways exist for community-based falls prevention: reactive (R), after a fall requiring medical attention; proactive (P), after professional referral of high-risk individuals; and self-referred (SR), voluntary intervention enrolment. The UK guidelines recommend scale-up of all three [‘recommended care’ (RC)], but scale-up of none [‘usual care’ (UC)], one (R, P, SR) or two (R+P, R+SR, P+SR) are potential options. This study aims to compare the options in terms of efficiency and equity.

**Methods:**

Cost-utility analysis from the societal perspective over a 40-year horizon identified the optimal strategy based on efficiency alone. Probabilistic sensitivity analysis accounted for parameter uncertainty. Efficiency and equity were jointly evaluated by distributional cost-effectiveness analysis. Alternative scenarios assessed changes in frailty, cognitive impairment, intervention demand and GP access.

**Results:**

Public sector cost-effectiveness threshold would need to exceed £30 000 per quality-adjusted life year (QALY) gained for RC to have the highest probability of being cost-effective. R and R+SR were cost-effective, with costs per QALY gained of £2365 (R versus UC) and £5516 (R+SR versus R). RC was cost-ineffective, incurring £34 258 per QALY gained versus R+SR. Other strategies were dominated. However, if decision-makers had the same relative health inequality aversion level as the English general public, RC was optimal in terms of efficiency and equity at threshold of £30 000 per QALY gained. Scenarios of worse geriatric health favoured RC.

**Conclusions:**

Both efficiency and relative health inequality need to be considered for the UK guideline-recommended falls prevention to be optimal versus other permutations of community-based strategies.

## Key Points

- Recommended falls prevention not likely cost-effective.
- Efficiency and equity considered together makes recommended falls prevention optimal.
- Falls prevention guidelines should consider efficiency and equity alongside clinical effectiveness.

## Background

Falls significantly impact geriatric health, inducing conditions such as fear of falling [1–3], depression [4] and functional dependence [5–8]. These conditions generate substantial costs for the health and social care systems [9–11] and for wider society in terms of productivity loss, private care expenditures and caregiver burden [12–14]. More than half of falls in older adults occur in the community setting [15]. Population ageing in the UK and globally heightens the need for falls prevention [16–18]. Availability of falls prevention interventions that have shown to be efficacious further motivates their widespread implementation [19–21].

There are established guidelines for community-based falls prevention [22–26]. In the UK, the clinical guideline 161 (CG161) issued by the National Institute for Health and Care Excellence (NICE) [22]—currently being updated [27]—offers normative guidance to practitioners and commissioners. CG161 focuses on the proactive (P) pathway initiated by older persons’ routine contact with care professionals: older persons are screened for falls risk based on falls history and gait/balance impairment, and if at high risk, referred to multifactorial intervention encompassing multidisciplinary risk assessment and tailored treatments. CG161 also incorporates the reactive (R) pathway, wherein older persons who experienced a fall requiring medical attention are referred to multifactorial intervention [22]. Another potential pathway is the self-referred (SR) pathway, wherein older persons enrol in a falls prevention intervention without direct professional referral [28, 29]. Indeed, much trial-based evidence concerns participants who self-refer [30–32]. Likewise, older persons perceive self-referral as the main point of access under current practice in the UK [33]. The three pathways operate in parallel and are nonmutually exclusive commissioning options.

The authors have previously developed a simulation model [34] to evaluate the efficiency and equity of the ‘recommended care’ (RC) scenario, representing the strategy recommended by CG161 and other UK guidelines [22, 28, 29], compared to the ‘usual care’ (UC) scenario, representing the current practice in Sheffield which was assumed to be a representative UK urban local health economy [34]. RC involves the concurrent scale-up of all three pathways to recommended levels. Yet this is clearly not the only commissioning option: the decision-maker could scale up only one or two of the three pathways. Moreover, previous analysis [34] found significant heterogeneity in service user characteristics between the pathways, suggesting that their performance would differ in terms of efficiency (i.e. cost-effectiveness) and equity [i.e. reduction in health inequalities, deemed unfair, between groups delineated by socioeconomic status (SES) quartile].

This study aims to evaluate the relative performance of scaling up one, two or all of the R, P and SR falls prevention pathways in the UK community setting, using the same community-based falls prevention model that previously evaluated RC relative to UC. The strategies are UC, R, P, SR, R+P, R+SR, P+SR and RC. These are compared in terms of cost-effectiveness evaluated via cost-utility analysis (CUA) from the societal perspective then together with equity via distributional cost-effectiveness analysis.

## Methods

This study adhered to the Consolidated Health Economic Evaluation Reporting Standards 2022 (CHEERS 2022) guidance [35] (see Appendix 1 in the Supplementary Data).

### Model overview

Comprehensive documentation on the development and base case analysis results (RC versus UC) for the community-based falls prevention model is presented elsewhere [34]. The model was conceptualised in Sheffield as a representative UK urban local health economy. The model type is discrete individual simulation with annual cycles [36]. The target population is community-dwelling adults older than 60 years with and without falls history at baseline.

Simulated individuals have the following characteristics: age, sex, SES quartile, falls history in the previous year, frailty index (range 0–100), physical activity (binary high versus low), cognitive impairment (binary), fear of falling (binary) and abnormal gait/balance (binary). Combinations of these characteristics influence their falls risk and intervention eligibility. In each annual cycle, individuals can access one of three falls prevention pathways—R, P or SR—depending on their eligibility and implementation factors (supply and demand). These access conditions are detailed in Appendices 2–4 in the Supplementary Data. After intervention receipt (if any), individuals face risks of fatal fall or other-cause mortality. Others face risks of nonfatal falls estimated via multivariate logistic regressions. There are six nonfatal faller types: no fall, single fall not requiring medical attention (non-MA fall), single fall requiring medical attention (MA fall), recurrent non-MA falls and recurrent falls with one or more MA fall(s). Each MA fall carried 28% chance of hospitalisation [29]. Falls directly incur healthcare costs and acute quality-adjusted life year (QALY) loss, with the levels of costs and loss depending on the most prevalent injury for each fall type (e.g. hip fracture for hospitalised MA fall) [37].

Falls also affect the trajectory of frailty progression to generate longer-term impacts [38], including the risk of long-term care (LTC) admission and changes in time-varying characteristics (e.g. physical activity) and outcomes. The latter include EQ-5D-3L; public sector primary and secondary healthcare and community care costs, including prescription costs; out-of-pocket care expenditure; paid and unpaid productivity value; and informal care cost. There are corresponding intervention costs: public sector expenditure, private copayment and time opportunity costs for participants and informal caregivers. Individuals exit at mortality, LTC admission or at final cycle, and discounted lifetime outcomes are calculated.

### Evaluated falls prevention strategies

The following falls prevention strategies are evaluated in this study:

1. UC
2. R pathway at recommended level
3. P pathway at recommended level
4. SR pathway at recommended level
5. R+P pathways at recommended level
6. R+SR pathways at recommended level
7. P+SR pathways at recommended level
8. RC

It was assumed that no supply-side constraint exists under recommended levels, i.e. all eligible persons would receive the intervention pending demand.

### Model analysis methods

#### Societal cost-utility analysis

As primary analysis, the study conducted CUA, using QALYs as the health outcome, from the societal perspective over 40-year horizon. The societal perspective enabled the analysis to capture a wider set of costs averted by falls prevention, including productivity loss, private care expenditure and informal caregiver burden. The 40-year horizon was a near-lifetime horizon for the initial cohort aged 60 years and over at model baseline, although new cohorts aged 60 years entered each year. The analysis thereby captured the relevant long-term consequences of falls prevention.

The ‘quality’ within QALY was measured by EQ-5D-3L. The societal perspective accounted for costs incurred outside of public sector and nonhealth outcomes (e.g. productivity value). Costs were reported in pounds (£) at year 2023 price. Both costs and health outcomes were discounted at 3.5% annually [39]. Distinction was made between all-cause costs and directly fall-related costs, as recommended [40]. Commonly used cost-effectiveness thresholds of £20 000–£30 000 per QALY gained were used to express the health opportunity cost of public sector costs [39], and £60 000 per QALY gained for societal costs [41]. The latter was used to convert the incremental nonpublic sector costs to their QALY equivalent which were then added to the QALY gains to obtain total societal QALY gains. An incremental cost-effectiveness ratio (ICER) was calculated as incremental public sector cost per societal QALY gained.

Efficiency was also reported as net benefits: incremental net monetary benefit (INMB), calculated by translating the societal QALY gained into monetary amount using the cost-effectiveness threshold and subtracting the incremental public sector cost, and incremental net health benefit (INHB), calculated by translating the incremental public sector cost into the QALY equivalent and adding to the societal QALY gained. INMB/INHB above 0 indicates efficiency gain.

#### Handling parameter uncertainty

The primary analysis reported probabilistic outcomes that account for second-order uncertainty around the point estimates of model input parameters [42, 43]. This was done by reporting the expected outcomes (i.e. averages) from the probabilistic sensitivity analysis (PSA) simulation runs. The distributions for the input parameters are described elsewhere (Section B9 of Appendix B) [34]. The societal ICER of RC versus UC previously stabilised after around 600 simulation runs [34]. Hence, 800 runs were performed for each strategy. A scatter plot of the incremental societal QALY and public sector cost for each run was presented. The cost-effectiveness acceptability curve (CEAC), depicting the probability of each strategy being the most cost-effective strategy at different public sector cross-effectiveness thresholds, was presented alongside the cost-effectiveness acceptability frontier (CEAF), which marks the strategy with the highest expected net monetary benefit (NMB) across runs at different intervals of the cost-effectiveness threshold [42].

#### Distributional cost-effectiveness analysis

Outcome differences across SES quartiles were deemed unfair. Distributional cost-effectiveness analysis (DCEA) was used to jointly consider the efficiency and equity impacts of intervention strategies [44], where equity is defined as reducing the relative or absolute outcome gap across SES quartiles. The outcome of interest was the per-capita societal net health benefit (NHB) accruing by subgroup. The equally distributed equivalent (EDE) level of societal NHB was calculated for each intervention strategy [44]. Atkinson index *ε* and Kolm index *α* depicted the strength of relative and absolute inequality aversions, respectively, where higher values denote greater aversion (ε = 0 implies no aversion); ε = 11 and α = 0.15 were elicited from the general public in England [44]. Overall, the strategy with the highest EDE level of societal NHB is the optimal one based on both efficiency and equity. Another key metric is the incremental EDE NHB (EDE INHB) of a given strategy versus a comparator. EDE INHB above 0 implied the given strategy should be preferred over the comparator based on efficiency and equity. For all DCEA outcomes, the expected value across the PSA simulation runs was presented at different levels of *ε* and *α*.

#### Alternative scenario analyses

The following alternative scenarios were assessed.

1) Varying frailty: (i) baseline frailty increased/decreased by 20% and (ii) rate of frailty progression between model cycles increased/decreased by 20%.
2) Incidence rate of cognitive impairment between cycles increased/decreased by 20%.
3) Demand rates for interventions by eligible persons increased/decreased by 20% (rates under UC remained unchanged).
4) GP access rate increased/decreased by 20%.

For each scenario, deterministic outcomes were assessed that accounted for first-order uncertainty arising from variability in simulated individuals [43]. This was done by re-running each analysis with 10 random number seeds and computing the average. Outcomes were reported under the DCEA framework concerning relative inequality aversion.

## Results

### Societal cost-utility analysis

Table 1 shows the incremental societal QALYs and public sector costs (all-cause) of the strategies relative to UC and the ICERs relative to the next best comparator. Appendix 5 in the Supplementary Data reports the absolute disaggregated outcomes. No strategy dominated UC by generating both cost savings and QALY gains. Strategies SR, P, R+P and P+SR were strongly or extendedly dominated other strategies. The ICERs between nondominated strategies were £2365 per QALY gained for R versus UC, £5516 per QALY gained for R+SR versus R and £34 258 per QALY gained for RC versus R+SR. At the public sector cost-effectiveness threshold of £30 000 per QALY gained and considering efficiency alone, the optimal strategy is likely to be R+SR.

**Table 1:** Incremental outcomes of strategies under 40-year societal CUA.

Strategy^a^	Incremental public sector cost (all-cause)^b^	Incremental societal QALY^b^	ICER (£ per QALY gained)	Comparator
R	£5 609 678	2371.6	£2365	UC
SR	£54 293 886	9915.1	Extendedly dominated	N/A
R+SR	£60 993 792	12413.0	£5516	R
P	£246 878 182	10386.3	Dominated	N/A
R+P	£249 448 665	12230.0	Dominated	N/A
P+SR	£280 980 595	17319.5	Extendedly dominated	N/A
RC	£284 401 514	18934.3	£34 258	R+SR

^a^Arranged in the order of increasing incremental public sector cost (all-cause).

^b^Incremental relative to the UC scenario. All outcomes are averages of outcomes from simulation runs under PSA.

**Figure 1:**
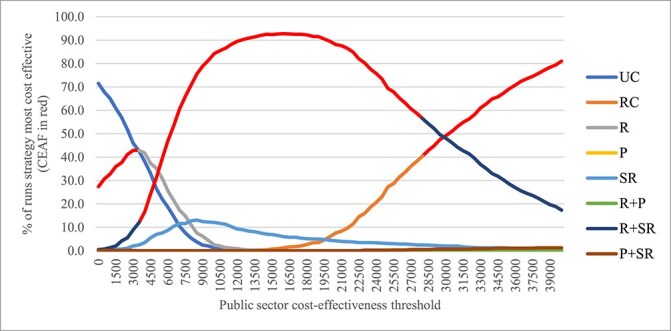
CEAC and CEAF. The societal cost-effectiveness threshold is held constant at £60 000 per QALY gained.

Appendix 6 in the Supplementary Data shows the scatter plot of the incremental outcomes versus UC. Figure 1 shows the CEAC and CEAF. When the public sector cost-effectiveness threshold was between £0 and £3500 per QALY gained, UC had the highest percentage of runs, wherein it was the most cost-effective strategy (42.9% at £3500 per QALY gained). However, over the same threshold interval, R had the highest expected NMB according to CEAF. For the threshold interval between £3500 and £28 000 per QALY gained, R+SR produced the highest expected NMB according to CEAF. Above £28 000 per QALY gained, RC had the highest expected NMB according to CEAF, although R+SR had a higher probability of being the most cost-effective strategy until the threshold reached £30 000 per QALY gained. Overall, when considering efficiency alone, the cost-effectiveness threshold would have to approach or exceed £30 000 per QALY gained for RC to be cost-effective versus R+SR, the next best comparator.

### Distributional cost-effectiveness analysis

**Figure 2:**
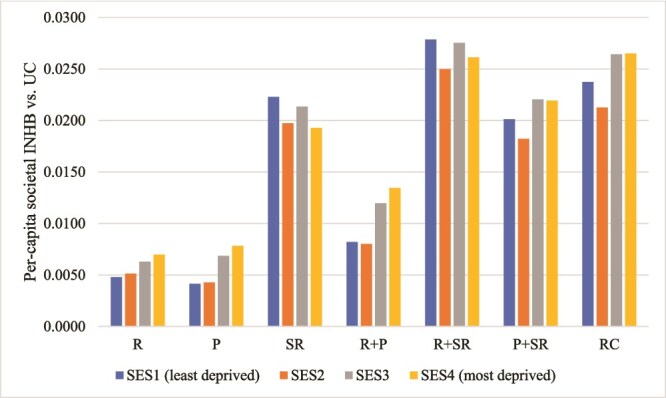
Average societal incremental NHB versus UC by strategy and SES quartile. Note: INHB calculated using public sector cost-effectiveness threshold of £30 000 per QALY gained.

Figure 2 shows the distribution of the per-capita societal INHB by SES quartile for each strategy versus UC at the public sector cost-effectiveness threshold of £30 000 per QALY gained. R and P were unambiguously progressive, with the magnitude of INHB increasing the more deprived the SES quartile. Other strategies were neither unambiguously progressive or regressive.

Table 2 shows the results of DCEA concerning relative inequality of per-capita societal NHB between SES quartiles. At the threshold of £20 000 per QALY gained, R+SR was consistently the optimal strategy in terms of both efficiency and equity across the range of Atkinson indices, including ε = 11 quantifying the aversion level of the English general public. At the higher threshold of £30 000 per QALY gained, R+SR was the optimal strategy under no (ε = 0) or low (ε = 5) aversion. Under higher aversion, including ε = 11, RC was optimal. This can be attributed to the P pathway within RC being unambiguously progressive.

**Table 2:** Result of distributional cost-effectiveness analysis concerning relative inequality between SES quartiles.

	Strategy^a^							
	UC	R	P	SR	R+P	R+SR	P+SR	RC
Public sector cost-effectiveness threshold = £20 000 per QALY gained								
EDE NHB (incremental EDE NHB versus UC)								
Atkinson index ε = 0	3.7127	3.7183 [0.0056]	3.7078 [−0.0049]	3.7311 [0.0184]	3.7124 [−0.0003]	3.7368* [0.0241]	3.7212 [0.0085]	3.7250 [0.0123]
ε = 5	3.1066	3.1146 [0.0080]	3.1033 [−0.0033]	3.1277 [0.0211]	3.1097 [0.0031]	3.1355* [0.0289]	3.1190 [0.0124]	3.1243 [0.0177]
ε=11^b^	2.7198	2.7278 [0.0080]	2.7170 [−0.0028]	2.7400 [0.0202]	2.7235 [0.0037]	2.7478* [0.0280]	2.7322 [0.0123]	2.7375 [0.0177]
ε = 15	2.6191	2.6269 [0.0078]	2.6164 [−0.0027]	2.6386 [0.0195]	2.6227 [0.0036]	2.6462* [0.0271]	2.6311 [0.0120]	2.6362 [0.0171]
ε = 20	2.5524	2.5600 [0.0076]	2.5498 [−0.0026]	2.5714 [0.0190]	2.5559 [0.0035]	2.5788* [0.0265]	2.5641 [0.0117]	2.5691 [0.0167]
ε = 30	2.4890	2.4964 [0.0074]	2.4865 [−0.0026]	2.5076 [0.0186]	2.4925 [0.0034]	2.5148* [0.0258]	2.5004 [0.0114]	2.5053 [0.0163]
Public sector cost-effectiveness threshold = £30 000 per QALY gained								
ε = 0	4.1808	4.1866 [0.0058]	4.1866 [0.0058]	4.2015 [0.0207]	4.1912 [0.0104]	4.2074* [0.0266]	4.2014 [0.0206]	4.2053 [0.0245]
ε = 5	3.7039	3.7114 [0.0075]	3.7121 [0.0082]	3.7261 [0.0222]	3.7181 [0.0142]	3.7335* [0.0296]	3.7282 [0.0243]	3.7331 [0.0292]
ε=11^b^	3.3219	3.3297 [0.0078]	3.3307 [0.0088]	3.3437 [0.0218]	3.3370 [0.0151]	3.3514 [0.0295]	3.3466 [0.0247]	3.3517* [0.0298]
ε = 15	3.2049	3.2126 [0.0077]	3.2135 [0.0086]	3.2262 [0.0212]	3.2197 [0.0148]	3.2337 [0.0287]	3.2291 [0.0241]	3.2341* [0.0291]
ε = 20	3.1245	3.1320 [0.0075]	3.1329 [0.0084]	3.1452 [0.0207]	3.1390 [0.0145]	3.1526 [0.0281]	3.1481 [0.0236]	3.1530* [0.0285]
ε = 30	3.0471	3.0545 [0.0073]	3.0554 [0.0082]	3.0674 [0.0202]	3.0613 [0.0141]	3.0746 [0.0274]	3.0702 [0.0230]	3.0750* [0.0278]

Abbreviation: EDE NHB, equally distributed equivalent per-capita societal net health benefit.

^a^Optimal strategy marked by * and shaded in grey.

^b^Atkinson index elicited from the general public in England [44].

Appendix 7 in the Supplementary Data shows the results of DCEA concerning absolute inequality of outcomes. R+SR remained optimal across both thresholds (£20 000/£30 000 per QALY gained) and the range of Kolm indices. Overall, there is some evidence that RC is the optimal strategy provided that the cost-effectiveness threshold is £30 000 per QALY gained or higher and the decision-maker has relative inequality aversion akin to that elicited from the English general public.

### Alternative scenario analyses

Table 3 shows the DCEA results for scenarios wherein the baseline frailty level has been increased and decreased by 20%. Compared to the main scenario, 20% increase in baseline frailty favoured RC by increasing the range of cost-effectiveness threshold and ε under which it is optimal. RC was now optimal under the threshold of £20 000 per QALY gained and aversion levels between ε = 11 and ε = 30. Decrease in baseline frailty also favoured RC but not to the same extent.

**Table 3:** Scenarios of change in baseline frailty.

Scenario: 20% increase in baseline frailty								
	Strategy^a^							
	UC	R	P	SR	R+P	R+SR	P+SR	RC
Public sector cost-effectiveness threshold = £20 000 per QALY gained								
EDE NHB (incremental EDE NHB versus UC)								
Atkinson index ε = 0	3.3138	3.3197 [0.0059]	3.3101 [−0.0037]	3.3332 [0.0194]	3.3167 [0.0029]	3.3376* [0.0238]	3.3255 [0.0117]	3.3320 [0.0182]
ε=11^b^	2.2062	2.2110 [0.0048]	2.1994 [−0.0067]	2.2257 [0.0196]	2.2105 [0.0043]	2.2271 [0.0210]	2.2147 [0.0085]	2.2301* [0.0240]
ε = 30	2.0158	2.0203 [0.0044]	2.0097 [−0.0062]	2.0338 [0.0179]	2.0198 [0.0040]	2.0350 [0.0192]	2.0236 [0.0078]	2.0378* [0.0220]
Public sector cost-effectiveness threshold = £30 000 per QALY gained								
ε = 0	3.7899	3.7961 [0.0062]	3.7970 [0.0071]	3.8116 [0.0217]	3.8035 [0.0136]	3.8160 [0.0261]	3.8139 [0.0240]	3.8204* [0.0304]
ε = 11	2.8246	2.8303 [0.0057]	2.8308 [0.0062]	2.8465 [0.0219]	2.8411 [0.0165]	2.8478 [0.0232]	2.8468 [0.0222]	2.8616* [0.0369]
ε = 30	2.5845	2.5897 [0.0052]	2.5902 [0.0057]	2.6047 [0.0202]	2.5997 [0.0152]	2.6058 [0.0213]	2.6048 [0.0204]	2.6186* [0.0341]
Scenario: 20% decrease in baseline frailty								
Public sector cost-effectiveness threshold = £20 000 per QALY gained								
ε = 0	4.1119	4.1169 [0.0050]	4.1083 [−0.0036]	4.1300 [0.0181]	4.1120 [0.0001]	4.1352* [0.0233]	4.1214 [0.0095]	4.1272 [0.0153]
ε = 11	3.2730	3.2791 [0.0061]	3.2706 [−0.0024]	3.2934 [0.0204]	3.2773 [0.0043]	3.3008* [0.0278]	3.2821 [0.0091]	3.2987 [0.0257]
ε = 30	3.0006	3.0063 [0.0057]	2.9984 [−0.0022]	3.0196 [0.0189]	3.0048 [0.0042]	3.0265* [0.0259]	3.0090 [0.0083]	3.0248 [0.0242]
Public sector cost-effectiveness threshold = £30 000 per QALY gained								
ε = 0	4.5700	4.5755 [0.0055]	4.5768 [0.0068]	4.5905 [0.0205]	4.5806 [0.0106]	4.5958 [0.0258]	4.5915 [0.0214]	4.5978* [0.0278]
ε = 11	3.8476	3.8545 [0.0069]	3.8567 [0.0090]	3.8699 [0.0223]	3.8632 [0.0156]	3.8770 [0.0294]	3.8685 [0.0208]	3.8866* [0.0390]
ε = 30	3.5397	3.5461 [0.0064]	3.5483 [0.0086]	3.5607 [0.0210]	3.5546 [0.0149]	3.5674 [0.0277]	3.5590 [0.0193]	3.5769* [0.0372]

^a^Optimal strategy marked by * and shaded in grey.

^b^Atkinson index elicited from the general public in England [44].

Appendices 8–9 in the Supplementary Data show the DCEA results for scenarios of increase and decrease in the rates of frailty progression and cognitive impairment incidence, respectively. The impacts on strategy ranking were similar to those of baseline frailty changes. Appendices 10–11 in the Supplementary Data show the results for scenarios of increase and decrease in the intervention demand and in the GP access rate, respectively.

## Discussion

This study comparatively assessed permutations of the R, P and SR falls prevention pathways in the UK community setting in terms of cost-effectiveness and reduction in social inequities of health. Under societal 40-year CUA accounting for efficiency alone, the RC strategy that significantly expands all three pathways is unlikely to be the optimal strategy unless the cost-effectiveness threshold approaches or exceeds £30 000 per QALY gained. However, if relative (although not absolute) inequality aversion of the decision-maker is as high as that of the English general public, RC would be optimal at the threshold of £30 000 per QALY gained. This can be attributed to its more progressive impact relative to the next best comparator (R+SR) which excludes the P pathway.

This study therefore provides a nuanced picture of whether the community-based falls prevention currently recommended by UK guidelines [22, 24, 28] is optimal in terms of efficiency and equity. First, previous model analysis [34] found RC to be highly cost-effective versus UC, with an ICER of £14 067 per QALY gained and 93.4% probability of being cost-effective at the cost-effectiveness threshold of £20 000 per QALY gained. However, the current study shows that the introduction of other pathway permutations no longer makes RC an attractive choice, with an ICER relative to R+SR exceeding £30 000 per QALY gained. That said, second, the finding that RC becomes optimal when relative inequality aversion is considered alongside efficiency increases its viability for decision-makers who consider equity. NICE, for instances, states that it would ‘take into account inequalities arising from socioeconomic factors’ and aim to ‘reduce and not increase identified health inequalities’ (Paragraph 29) [45]. Nevertheless, how NICE would choose between strategies that each reduce health inequalities, although to different extents (as was the case for RC and R+SR), remains unspecified. DCEA can guide such decisions [44] but is not yet, to our knowledge, part of any health technology assessment guidance.

Third, the finding that R+SR is the optimal strategy across wide ranges of threshold and inequality aversion levels challenges the significant emphasis on the P pathway placed by the NICE falls prevention guideline [22]. It should also be noted that the emphasis on the P pathway is found in falls prevention guidelines of other countries: specifically, 13 of 15 guidelines identified in a systematic review strongly recommended risk screening based on falls history, gait/balance difficulties and fear of falling [25]. Therefore, the model finding challenging the relative performance of the P pathway is likely transferable to non-UK settings with the above recommendation. At the least, the guidelines should clarify that the comparative evaluation becomes nuanced when efficiency and equity are considered alongside clinical effectiveness.

This study possesses some key strengths. First, it utilised a previously validated health economic model [34]. Second, it jointly explored efficiency and equity using DCEA, which contrasts with the scarcity of attempts to account for equity by existing models [46]. There are nevertheless limitations. First, the DCEA used SES quartile as the sole subgroup delineator of equity relevance. However, earlier conceptual work identified further equity-relevant delineators such as living alone [33]. Second, more alternative scenarios could have been evaluated, including differing discount rates, time horizons and other intervention features such as costs. Incorporating capacity constraints in the model is an aspect warranting further research [47, 48]. There are clear limits on the resources for community-based falls prevention (e.g. number of multidisciplinary falls clinics) that can be accommodated in any local health economy; however, the current scenarios did not characterise such constraints. One important constraint is the burden placed on primary care from the scale-up of the P pathway. The model assumed that under the scale-up all GP consultations would incorporate falls risk screening [34], but this may not be feasible in practice. Finally, the model focused on evaluating the recommendations of UK falls prevention guidelines and may thus lack external validity to non-UK settings—although as noted, the emphasis on the P pathway is shared by UK and non-UK guidelines.

## Conclusion

Compared to scaling up one or two of the R, P and SR pathways, concurrently scaling up all three as recommended by the UK guidelines would be optimal in terms of efficiency and social equity only if the decision-makers are willing to pay just over £30 000 per QALY gained and accept the relative inequality aversion is at the level elicited by the English general public. Increased frailty in the older population would favour the recommended strategy. Otherwise, the optimal strategy is to scale up R and SR pathways only.

## Supplementary Material

aa-24-2551-File002_afaf212

## Data Availability

The documents informing the model conceptualisation, the Simul8 model file and the model outputs generated by the current study are available from the corresponding author on reasonable request.
