# Supplementary material for: Efficiency and equity of community-based falls prevention pathways: a model-based health economic evaluation
Source: Age Ageing. 2025 Aug 3;54(8):afaf212. doi: 10.1093/ageing/afaf212 (PMC12318474; doi:10.1093/ageing/afaf212)
Supplement: aa-24-2551-File002_afaf212 [file aa-24-2551-file002_afaf212.docx]

# Efficiency and equity of community-based falls prevention pathways: a model-based health economic evaluation

**Supplementary Data**

**Keywords:** falls prevention; economic model; NICE falls prevention guideline; equity

Contents

[Efficiency and equity of community-based falls prevention pathways: a model-based health economic evaluation 1](#_Toc202515720)

[CHEERS 2022 checklist (Appendix 1) 2](#_Toc202515721)

[Intervention access conditions 6](#_Toc202515722)

[Societal cost-utility analysis 9](#_Toc202515723)

[Distribution cost-effectiveness analysis 11](#_Toc202515724)

[Scenario analyses 12](#_Toc202515725)

[References 16](#_Toc202515726)

# CHEERS 2022 checklist (Appendix 1)

| **Topic** | **No.** | **Item** | **Location where item is reported** |
| --- | --- | --- | --- |
| **Title** |  |  |  |
|  | 1 | Identify the study as an economic evaluation and specify the interventions being compared. | Title |
| **Abstract** |  |  |  |
|  | 2 | Provide a structured summary that highlights context, key methods, results, and alternative analyses. | Abstract |
| **Introduction** |  |  |  |
| **Background and objectives** | 3 | Give the context for the study, the study question, and its practical relevance for decision making in policy or practice. | 'Background' |
| **Methods** |  |  |  |
| **Health economic analysis plan** | 4 | Indicate whether a health economic analysis plan was developed and where available. | Reference to how the decision problem was conceptualised is provided in 'Model overview' |
| **Study population** | 5 | Describe characteristics of the study population (such as age range, demographics, socioeconomic, or clinical characteristics). | 'Model overview' |
| **Setting and location** | 6 | Provide relevant contextual information that may influence findings. | 'Model overview' |
| **Comparators** | 7 | Describe the interventions or strategies being compared and why chosen. | 'Evaluated falls prevention strategies' |
| **Perspective** | 8 | State the perspective(s) adopted by the study and why chosen. | 'Societal cost-utility analysis' |
| **Time horizon** | 9 | State the time horizon for the study and why appropriate. | 'Societal cost-utility analysis' |
| **Discount rate** | 10 | Report the discount rate(s) and reason chosen. | 'Societal cost-utility analysis' |
| **Selection of outcomes** | 11 | Describe what outcomes were used as the measure(s) of benefit(s) and harm(s). | 'Model overview' and 'Societal cost-utility analysis' |
| **Measurement of outcomes** | 12 | Describe how outcomes used to capture benefit(s) and harm(s) were measured. | 'Model overview' and 'Societal cost-utility analysis' |
| **Valuation of outcomes** | 13 | Describe the population and methods used to measure and value outcomes. | 'Model overview' and 'Societal cost-utility analysis' |
| **Measurement and valuation of resources and costs** | 14 | Describe how costs were valued. | 'Model overview' and 'Societal cost-utility analysis' |
| **Currency, price date, and conversion** | 15 | Report the dates of the estimated resource quantities and unit costs, plus the currency and year of conversion. | 'Societal cost-utility analysis' |
| **Rationale and description of model** | 16 | If modelling is used, describe in detail and why used. Report if the model is publicly available and where it can be accessed. | 'Model overview' |
| **Analytics and assumptions** | 17 | Describe any methods for analysing or statistically transforming data, any extrapolation methods, and approaches for validating any model used. | 'Model overview', Tables A1-A3 |
| **Characterising heterogeneity** | 18 | Describe any methods used for estimating how the results of the study vary for subgroups. | 'Distributional cost-effectiveness analysis' |
| **Characterising distributional effects** | 19 | Describe how impacts are distributed across different individuals or adjustments made to reflect priority populations. | 'Distributional cost-effectiveness analysis' |
| **Characterising uncertainty** | 20 | Describe methods to characterise any sources of uncertainty in the analysis. | 'Handling parameter uncertainty' and 'Alternative scenario analyses' |
| **Approach to engagement with patients and others affected by the study** | 21 | Describe any approaches to engage patients or service recipients, the general public, communities, or stakeholders (such as clinicians or payers) in the design of the study. | Reference to how the decision problem and the model structure were conceptualised with stakeholder inputs is provided in 'Model overview' |
| **Results** |  |  |  |
| **Study parameters** | 22 | Report all analytic inputs (such as values, ranges, references) including uncertainty or distributional assumptions. | Reference to previous analysis that documented the analytic inputs is provided in 'Model overview' |
| **Summary of main results** | 23 | Report the mean values for the main categories of costs and outcomes of interest and summarise them in the most appropriate overall measure. | 'Societal cost-utility analysis' under Results for evaluation considering efficiency alone. 'Distributional cost-effectiveness analysis' under Results for evaluation considering efficiency and equity. |
| **Effect of uncertainty** | 24 | Describe how uncertainty about analytic judgments, inputs, or projections affect findings. Report the effect of choice of discount rate and time horizon, if applicable. | Parameter uncertainty is accounted for in all analyses reported in 'Societal cost-utility analysis' and 'Distributional cost-effectiveness analysis' under Results. Results of scenario analyses are reported in 'Alternative scenario analyses' |
| **Effect of engagement with patients and others affected by the study** | 25 | Report on any difference patient/service recipient, general public, community, or stakeholder involvement made to the approach or findings of the study | Reference to how the decision problem and the model structure were conceptualised with stakeholder inputs is provided in 'Model overview' |
| **Discussion** |  |  |  |
| **Study findings, limitations, generalisability, and current knowledge** | 26 | Report key findings, limitations, ethical or equity considerations not captured, and how these could affect patients, policy, or practice. | 'Discussion' |
| **Other relevant information** |  |  |  |
| **Source of funding** | 27 | Describe how the study was funded and any role of the funder in the identification, design, conduct, and reporting of the analysis | 'Declarations' |
| **Conflicts of interest** | 28 | Report authors conflicts of interest according to journal or International Committee of Medical Journal Editors requirements. | 'Declarations' |

*From:* Husereau D, Drummond M, Augustovski F, et al. Consolidated Health Economic Evaluation Reporting Standards 2022 (CHEERS 2022) Explanation and Elaboration: A Report of the ISPOR CHEERS II Good Practices Task Force. Value Health 2022;25. <doi:10.1016/j.jval.2021.10.008>

# Intervention access conditions

| **Appendix 2** Falls prevention intervention eligibility conditions by pathway and scenario. | | | |
| --- | --- | --- | --- |
| **Pathway** |  | **Usual care (UC)** | **Recommended care (RC)** |
| **Reactive** | Eligibility | HAM for hospitalised fallers only (around 28% of MA fallers). | Multifactorial intervention for all MA fallers.^1^ |
|  | Access rate | Frailty gradient to access among MA fallers: 9.6% if Fit; 23.5% if Mild frailty; 51.7% if Moderate; 72.7% if Severe; 28.7% overall.^2^ | All eligible persons pending demand: 53.8% uptake rate for cognitively intact [1]; 49.6% for cognitively impaired [2] |
| **Proactive** | Eligibility | Multifactorial intervention for high falls risk persons screened at routine GP contact, who are: (i) cognitively intact; (ii) not receiving the reactive intervention that year; (iii) have not previously received the proactive intervention. | Persons at high falls risk^3^ screened at routine GP contract, who are not receiving the reactive intervention that year; intervention type varying by cognitive status:  (1) Multifactorial intervention for cognitively intact persons, up to three receipts.^4^  (2) Tai Chi for cognitively impaired regardless of intervention history. |
|  | Access rate | (A) *Routine GP contact*: 81.3% of persons aged 60+ access GP each year.^5^  (B) *Falls risk screening*: frailty gradient to access among those who received GP contact – 12.0% if Fit; 15.1% if Mild; 19.7% if Moderate; 21.9% if Severe; 15.7% overall.^6^  (C) *Intervention*: frailty gradient to access among those screened at GP contact and cognitively intact – 10.9% if Fit; 24.7% if Mild; 53.6% if Moderate; 100% if Severe; 33.5% overall.^2^ | (A) *Routine GP contact*: 81.3% of persons aged 60+ access GP each year.^5^  (B) *Falls risk screening*: 100% access among those who received GP contact.  (C) *Intervention*: all eligible persons pending demand – 82.4% for multifactorial intervention for cognitively intact persons [3]; 44.5% for Tai Chi for cognitively impaired persons [4]. |
| **Self-referred** | Eligibility | Self-financed exercise intervention (FaME and Otago) for all persons not receiving the reactive/proactive intervention that year. | Publicly funded exercise intervention (FaME and Otago) for all persons not receiving the reactive/proactive intervention that year. |
|  | Access rate | 0.1% of persons in the most privileged SES quartile (around 50 persons per year). | All eligible persons pending demand: uptake rate of 9.5% overall with variation by individual-level characteristics.^7^ |
| **Abbreviation:** ELSA: English Longitudinal Study of Ageing; FaME: Falls Management Exercise; HAM: home assessment and modification; MA faller: faller requiring medical attention; NICE: National Institute for Health and Care Excellence; SES: socioeconomic status.  ^1^ According to NICE CG161.  ^2^ According to analysis of ELSA Wave 4 data.  ^3^ According to NICE CG161 criteria: high falls risk if had recurrent falls in past year and/or abnormal gait/balance [5].  ^4^ To keep client flow to that compatible with seven falls clinics, those with three previous receipts are excluded.  ^5^ See Table A2 for logistic regression result predicting routine GP access based on individual-level characteristics (e.g., age, sex, frailty, cognitive impairment).  ^6^ According to analysis of ELSA Wave 4 data. These percentages are proportions of persons with recurrent non-MA or MA falls history. Persons with no falls history or single non-MA fall history did not receive screening according to ELSA.  ^7^ See Table A3 for logistic regression result predicting self-referred exercise demand based on individual-level characteristics (e.g., age, sex, frailty, physical activity level). | | | |

How the conditions and rates were conceptualised and parameterised, as well as the costs and efficacies of the interventions, are detailed in section A7 of Appendix A and section B5 of Appendix B of the previous publication on the falls prevention model [6].

The reactive pathway would see a shift from the provision of home assessment and modification (HAM) only for hospitalised fallers under UC to the provision of multifactorial intervention for all MA fallers (pending demand) under RC.

The proactive pathway contains three components: routine GP contact; falls risk screening; and intervention for those at high falls risk. The annual probability of routine GP contact, estimated from ELSA, was assumed unchanged across UC and RC. Under RC, the rate of falls risk screening at GP contact would see a substantial increase from 15.7% of those with histories of recurrent non-MA falls or MA fall(s) under UC (ELSA data showed no screening receipt among those without such histories) to 100% of those receiving routine GP contact. The type of proactive intervention depended on cognitive status and history of intervention receipt. Under UC, multifactorial intervention was assumed indicated only for cognitively intact persons; those with cognitive impairment would be referred to the memory clinic instead. By contrast, cognitively impaired persons were assumed indicated for a proactive intervention under RC. In the absence of any UK-based RCT evidence on proactive multifactorial intervention (such as [3] for cognitively intact persons), cognitively impaired persons were assumed to receive Tai Chi tailored to this subgroup [4]. Under UC, those who have already received the proactive multifactorial intervention were assumed ineligible for re-receipt. Under RC, eligible persons were allowed up to three receipts to keep the client flow to multidisciplinary falls clinics to that compatible with the capacity of seven full-time clinics (around 21,000 clients per year).

According to stakeholder discussions [7], the self-referred pathway under current practice (represented by UC) comprised a small number of self-financing members of group exercise in a well-to-do neighbourhood of Sheffield. Under RC, access to publicly funded group exercise would expand to the rate of group exercise uptake as predicted using ELSA (9.5% of persons aged 60+). This rate was broadly comparable to the uptake rate in an RCT of group and home exercise wherein older persons were invited (rather than professionally referred) to participate [8].

| **Appendix 3** Logistic regression coefficients for GP routine contact. | | |
| --- | --- | --- |
| ***Dependent variable: GP routine contact*** | | |
| **Explanatory variables** | **Coefficient (SE)** | **P-value** |
| Constant | -20.065 (3.041) | <0.001 |
| Age, previous cycle (PC) | 0.568 (0.085) | <0.001 |
| Age^2, PC | -0.004 (0.0006) | <0.001 |
| Female | -0.192 (0.079) | 0.015 |
| Falls incidence (ref: No fall incidence) |  |  |
| *Single non-MA fall* | 0.269 (0.127) | 0.034 |
| *Recurrent non-MA falls* | 0.491 (0.165) | 0.003 |
| *Single MA fall* | 0.973 (0.247) | <0.001 |
| *Recurrent falls with MA* | 0.933 (0.364) | 0.010 |
| Frailty, PC (0-100) | 0.132 (0.013) | <0.001 |
| Frailty^2, PC | -0.002 (0.0003) | <0.001 |
| Change in frailty^1^ | 0.038 (0.008) | <0.001 |
| Cognitive impairment, PC | -0.692 (0.095) | <0.001 |
| Abnormal gait/balance, PC | -0.329 (0.121) | 0.007 |
| Social care receipt, PC | -0.785 (0.302) | 0.009 |
| Informal care receipt, PC | -0.255 (0.120) | 0.034 |
| GP routine contact, PC | 1.799 (0.081) | <0.001 |
| **Abbreviation:** ELSA: English Longitudinal Study of Ageing; MA fall: fall requiring medical attention; PC: previous cycle; Ref: reference; SE: standard error  ^1^ Covariate included in logistic regression is biannual change in frailty measured in ELSA. In model simulation, the annualized change in frailty is used instead to predict dependent variable. | | |

| **Appendix 4** Logistic regression coefficients for self-referred exercise demand. | | |
| --- | --- | --- |
| ***Dependent variable: Self-referred exercise demand*** | | |
| **Explanatory variables** | **Coefficient (SE)** | **P-value** |
| Constant | -3.211 (0.464) | <0.001 |
| Age, previous cycle (PC) | -0.017 (0.006) | 0.010 |
| Female | 0.627 (0.096) | <0.001 |
| Falls incidence (ref: No fall incidence) |  |  |
| *Single non-MA fall* | 0.184 (0.137) | 0.179 |
| *Recurrent non-MA falls* | 0.264 (0.148) | 0.075 |
| *Single MA fall* | 0.371 (0.191) | 0.052 |
| *Recurrent falls with MA* | 0.490 (0.212) | 0.021 |
| Frailty, PC (0-100) | 0.069 (0.015) | <0.001 |
| Frailty^2, PC | -0.0008 (0.0003) | 0.013 |
| Change in frailty^1^ | 0.040 (0.007) | <0.001 |
| High physical activity, PC | 0.590 (0.115) | <0.001 |
| Abnormal gait/balance, PC | -0.253 (0.129) | 0.050 |
| OOP care receipt, PC | 0.480 (0.208) | 0.021 |
| Exercise receipt, PC | 1.812 (0.107) | <0.001 |
| **Abbreviation:** ELSA: English Longitudinal Study of Ageing; MA fall: fall requiring medical attention; OOP: out-of-pocket; PC: previous cycle; Ref: reference; SE: standard error  ^1^ Covariate included in logistic regression is two-year change in frailty measured in ELSA. In model simulation, the annualised change in frailty is used instead to predict dependent variable. | | |

# Societal cost-utility analysis

| **Appendix 5** Probabilistic outcomes under 40-year societal cost-utility analysis. | | | | | | | | |
| --- | --- | --- | --- | --- | --- | --- | --- | --- |
| N=385,192 | **Usual care** | **Reactive (R)** | **Proactive (P)** | **Self-referred (SR)** | **R + P** | **R + SR** | **P + SR** | **Recommended care** |
| **Public sector cost^1^** | Mean (SD) | Mean (SD) | Mean (SD) | Mean (SD) | Mean (SD) | Mean (SD) | Mean (SD) | Mean (SD) |
| *All-cause public sector costs^2^* | £10,652,732,207 (£1,078,188,305) | £10,628,279,211 (£1,074,696,348) | £10,582,258,991 (£1,061,474,723) | £10,604,278,887 (£1,067,224,965) | £10,560,799,393 (£1,058,843,408) | £10,582,807,944 (£1,062,686,206) | £10,537,536,140 (£1,053,319,302) | £10,518,101,846 (£1,047,696,285) |
| *Fall-related healthcare cost* | £700,650,926 (£204,645,447) | £680,029,139 (£198,112,153) | £642,360,202 (£187,618,279) | £663,657,046 (£193,828,512) | £624,500,688 (£182,373,053) | £644,998,680 (£188,173,262) | £608,219,169 (£177,961,894) | £592,515,978 (£173,159,311) |
| *Public sector intervention cost* | £24,046,220 (£6,550,300) | £41,457,524 (£8,695,174) | £184,087,968 (£26,731,689) | £127,109,949 (£13,126,970) | £198,727,784 (£31,480,700) | £143,067,618 (£16,180,812) | £263,874,245 (£30,041,285) | £277,764,767 (£35,549,164) |
| **QALY** | 2,092,189 (56,202) | 2,094,338 (57,826) | 2,102,417 (56,171) | 2,101,062 (56,412) | 2,104,065 (57,267) | 2,103,347 (56,335) | 2,108,655 (58,310) | 2,110,096 (60,590) |
| **Societal outcome** |  |  |  |  |  |  |  |  |
| Productivity |  |  |  |  |  |  |  |  |
| *Productivity value^3^* | £15,409,567,554 (£3,126,414,242) | £15,412,082,742 (£3,131,754,175) | £15,433,605,114 (£3,130,030,118) | £15,433,569,287 (£3,129,326,013) | £15,434,432,153 (£3,134,612,753) | £15,438,861,403 (£3,131,558,777) | £15,446,057,408 (£3,135,451,897) | £15,444,371,061 (£3,132,711,691) |
| *Intervention TOC* | £1,436,988 (£854,033) | £3,365,578 (£1,030,727) | £32,223,961 (£7,641,937) | £24,420,561 (£4,940,558) | £32,708,712 (£7,628,996) | £25,950,648 (£5,237,224) | £50,826,472 (£11,012,582) | £51,169,747 (£10,928,182) |
| Personal finance |  |  |  |  |  |  |  |  |
| *OOP care expenditure^4^* | £2,762,610,945 (£612,689,051) | £2,757,170,040 (£611,974,366) | £2,736,499,740 (£608,486,991) | £2,736,391,535 (£607,850,717) | £2,731,446,907 (£606,358,553) | £2,731,781,536 (£607,405,087) | £2,718,065,715 (£604,625,012) | £2,713,360,194 (£603,041,281) |
| *Intervention co-payment* | £9,617,694 (£3,454,610) | £15,896,216 (£4,095,419) | £57,972,310 (£11,217,272) | £34,316,790 (£6,097,055) | £63,036,721 (£13,343,387) | £40,136,646 (£7,831,978) | £77,655,008 (£15,013,136) | £82,394,052 (£16,815,183) |
| Informal care |  |  |  |  |  |  |  |  |
| *Caregiving cost* | £15,903,375,001 (£3,283,458,386) | £15,882,463,927 (£3,282,762,823) | £15,829,500,834 (£3,269,033,285) | £15,823,860,110 (£3,266,622,294) | £15,811,571,995 (£3,269,401,834) | £15,806,698,947 (£3,264,535,356) | £15,766,212,225 (£3,257,082,651) | £15,747,437,798 (£3,250,337,791) |
| *Intervention TOC for caregiver* | £387,148 (£116,650) | £7,691,774 (£2,376,425) | £35,765,593 (£11,099,487) | £19,864,005 (£4,230,277) | £42,275,961 (£12,782,934) | £26,826,304 (£5,828,773) | £49,915,244 (£12,534,249) | £56,182,762 (£14,037,709) |
| **Abbreviation:** OOP: out-of-pocket; QALY: quality-adjusted life year; SD: standard deviation; TOC: time opportunity cost.  ^1^ All outcomes were averaged across 800 model simulation runs with different draws of model input parameters under probabilistic sensitivity analysis.  ^2^ Costs of fall-related primary and secondary healthcare, comorbidity primary and secondary healthcare, cost of dying, community healthcare, short-term social care, all-cause long-term care.  ^3^ Includes monetary values of paid and unpaid employment.  ^4^ Includes OOP care expenditure and privately incurred long-term care cost. | | | | | | | | |

**Appendix 6** Scatter plot of the incremental outcomes versus usual care of all intervention strategies. **Abbreviation:** P: proactive; QALY: quality-adjusted life year; R: reactive; RC: recommended care; SR: self-referred; UC: usual care.

# Distribution cost-effectiveness analysis

| **Appendix 7** Result of distributional cost-effectiveness analysis concerning absolute inequality between socioeconomic status quartiles. | | | | | | | | |
| --- | --- | --- | --- | --- | --- | --- | --- | --- |
|  | **Strategy^1^** | | | | | | | |
|  | **UC** | **R** | **P** | **SR** | **R+P** | **R+SR** | **P+SR** | **RC** |
| **Public sector cost-effectiveness threshold = £20,000 per QALY gained** | | | | | | | | |
| Equally distributed equivalent per-capita societal net health benefit (EDE NHB) [incremental EDE NHB vs. UC] | | | | | | | | |
| Kolm index α=0.01 | 3.7091 | 3.7147  [0.0056] | 3.7042  [-0.0049] | 3.7275  [0.0184] | 3.7088  [-0.0003] | 3.7332*  [0.0241] | 3.7176  [0.0085] | 3.7214  [0.0123] |
| α=0.025 | 3.7037 | 3.7093  [0.0056] | 3.6988  [-0.0048] | 3.7221  [0.0184] | 3.7034  [-0.0003] | 3.7278*  [0.0241] | 3.7122  [0.0085] | 3.7160  [0.0124] |
| α=0.05 | 3.6946 | 3.7003  [0.0056] | 3.6898  [-0.0048] | 3.7130  [0.0184] | 3.6944  [-0.0002] | 3.7187*  [0.0241] | 3.7032  [0.0086] | 3.7070  [0.0124] |
| α=0.15^2^ | 3.6576 | 3.6633  [0.0057] | 3.6529  [-0.0047] | 3.6759  [0.0183] | 3.6576  [0.0000] | 3.6817*  [0.0241] | 3.6663  [0.0087] | 3.6702  [0.0126] |
| α=0.25 | 3.6196 | 3.6254  [0.0058] | 3.6150  [-0.0045] | 3.6378  [0.0183] | 3.6197  [0.0002] | 3.6437*  [0.0242] | 3.6284  [0.0088] | 3.6323  [0.0128] |
| α=0.5 | 3.5214 | 3.5274  [0.0060] | 3.5172  [-0.0041] | 3.5395  [0.0182] | 3.5221  [0.0007] | 3.5456*  [0.0242] | 3.5306  [0.0092] | 3.5346  [0.0132] |
| **Public sector cost-effectiveness threshold = £30,000 per QALY gained** | | | | | | | | |
| α=0.01 | 4.1776 | 4.1834  [0.0058] | 4.1834  [0.0058] | 4.1982  [0.0207] | 4.1880  [0.0104] | 4.2042*  [0.0266] | 4.1982  [0.0206] | 4.2021  [0.0245] |
| α=0.025 | 4.1727 | 4.1785  [0.0058] | 4.1785  [0.0058] | 4.1933  [0.0206] | 4.1831  [0.0105] | 4.1993*  [0.0266] | 4.1933  [0.0206] | 4.1972  [0.0245] |
| α=0.05 | 4.1645 | 4.1704  [0.0058] | 4.1704  [0.0058] | 4.1851  [0.0206] | 4.1750  [0.0105] | 4.1911*  [0.0266] | 4.1851  [0.0206] | 4.1891  [0.0245] |
| α=0.15^2^ | 4.1312 | 4.1371  [0.0059] | 4.1371  [0.0059] | 4.1517  [0.0205] | 4.1418  [0.0107] | 4.1578*  [0.0266] | 4.1519  [0.0207] | 4.1558  [0.0246] |
| α=0.25 | 4.0969 | 4.1029  [0.0060] | 4.1030  [0.0061] | 4.1174  [0.0205] | 4.1078  [0.0108] | 4.1235*  [0.0265] | 4.1177  [0.0208] | 4.1217  [0.0248] |
| α=0.5 | 4.0085 | 4.0146  [0.0062] | 4.0148  [0.0064] | 4.0287  [0.0203] | 4.0197  [0.0113] | 4.0349*  [0.0265] | 4.0294  [0.0209] | 4.0335  [0.0250] |
| **Abbreviation:** P: proactive; QALY: quality-adjusted life year; R: reactive; RC: recommended care; SR: self-referred; UC: usual care  ^1^ Optimal strategy marked by * and shaded in grey.  ^2^ Kolm index elicited from the general public in England [9]. | | | | | | | | |

# Scenario analyses

| **Appendix 8** Scenarios of change in rate of frailty progression. | | | | | | | | |
| --- | --- | --- | --- | --- | --- | --- | --- | --- |
| **Scenario: 20% increase in frailty progression rate** | | | | | | | | |
|  | **Strategy^1^** | | | | | | | |
|  | **UC** | **R** | **P** | **SR** | **R+P** | **R+SR** | **P+SR** | **RC** |
| **Public sector cost-effectiveness threshold = £20,000 per QALY gained** | | | | | | | | |
| Equally distributed equivalent per-capita societal net health benefit (EDE NHB) [incremental EDE NHB vs. UC] | | | | | | | | |
| Atkinson index ε=0 | 3.4471 | 3.4558  [0.0087] | 3.4486  [0.0015] | 3.4716  [0.0245] | 3.4549  [0.0078] | 3.4769*  [0.0298] | 3.4667  [0.0196] | 3.4722  [0.0251] |
| ε=11^2^ | 2.4568 | 2.4686  [0.0119] | 2.4620  [0.0052] | 2.4811  [0.0244] | 2.4705  [0.0137] | 2.4887  [0.0319] | 2.4780  [0.0213] | 2.4920*  [0.0353] |
| ε=30 | 2.2461 | 2.2570  [0.0109] | 2.2509  [0.0048] | 2.2685  [0.0223] | 2.2588  [0.0127] | 2.2754  [0.0293] | 2.2656  [0.0195] | 2.2786*  [0.0325] |
| **Public sector cost-effectiveness threshold = £30,000 per QALY gained** | | | | | | | | |
| ε=0 | 3.9203 | 3.9290  [0.0087] | 3.9321  [0.0118] | 3.9466  [0.0263] | 3.9384  [0.0181] | 3.9520  [0.0317] | 3.9514  [0.0311] | 3.9574*  [0.0370] |
| ε=11 | 3.0677 | 3.0792  [0.0115] | 3.0844  [0.0167] | 3.0928  [0.0251] | 3.0919  [0.0241] | 3.1001  [0.0324] | 3.0996  [0.0319] | 3.1148*  [0.0470] |
| ε=30 | 2.8104 | 2.8211  [0.0107] | 2.8260  [0.0156] | 2.8336  [0.0232] | 2.8329  [0.0225] | 2.8403  [0.0299] | 2.8398  [0.0294] | 2.8542*  [0.0438] |
| **Scenario: 20% decrease in frailty progression rate** | | | | | | | | |
| **Public sector cost-effectiveness threshold = £20,000 per QALY gained** | | | | | | | | |
| ε=0 | 4.0029 | 4.0075  [0.0046] | 3.9957  [-0.0072] | 4.0169  [0.0140] | 3.9994  [-0.0035] | 4.0224*  [0.0195] | 4.0067  [0.0038] | 4.0117  [0.0088] |
| ε=11 | 3.0325 | 3.0377  [0.0052] | 3.0228  [-0.0097] | 3.0474  [0.0149] | 3.0296  [-0.0029] | 3.0506*  [0.0181] | 3.0315  [-0.0010] | 3.0455  [0.0130] |
| ε=30 | 2.7759 | 2.7806  [0.0048] | 2.7669  [-0.0090] | 2.7896  [0.0137] | 2.7732  [-0.0027] | 2.7925*  [0.0166] | 2.7748  [-0.0011] | 2.7879  [0.0120] |
| **Public sector cost-effectiveness threshold = £30,000 per QALY gained** | | | | | | | | |
| ε=0 | 4.4636 | 4.4686  [0.0050] | 4.4669  [0.0033] | 4.4798  [0.0162] | 4.4707  [0.0071] | 4.4856*  [0.0220] | 4.4794  [0.0158] | 4.4848  [0.0213] |
| ε=11 | 3.6171 | 3.6232  [0.0061] | 3.6199  [0.0028] | 3.6346  [0.0176] | 3.6261  [0.0091] | 3.6373  [0.0202] | 3.6285  [0.0115] | 3.6435*  [0.0264] |
| ε=30 | 3.3194 | 3.3251  [0.0057] | 3.3220  [0.0026] | 3.3358  [0.0164] | 3.3279  [0.0084] | 3.3381  [0.0187] | 3.3298  [0.0104] | 3.3442*  [0.0247] |
| **Abbreviation:** P: proactive; QALY: quality-adjusted life year; R: reactive; RC: recommended care; SR: self-referred; UC: usual care  ^1^ Optimal strategy marked by * and shaded in grey.  ^2^ Atkinson index elicited from the general public in England [9]. | | | | | | | | |

| **Appendix 9** Scenarios of change in incidence rate of cognitive impairment. | | | | | | | | |
| --- | --- | --- | --- | --- | --- | --- | --- | --- |
| **Scenario: 20% increase in incidence of cognitive impairment** | | | | | | | | |
|  | **Strategy^1^** | | | | | | | |
|  | **UC** | **R** | **P** | **SR** | **R+P** | **R+SR** | **P+SR** | **RC** |
| **Public sector cost-effectiveness threshold = £20,000 per QALY gained** | | | | | | | | |
| Equally distributed equivalent per-capita societal net health benefit (EDE NHB) [incremental EDE NHB vs. UC] | | | | | | | | |
| Atkinson index ε=0 | 3.6556 | 3.6605  [0.0049] | 3.6534  [-0.0021] | 3.6732  [0.0176] | 3.6562  [0.0006] | 3.6756*  [0.0200] | 3.6658  [0.0102] | 3.6690  [0.0134] |
| ε=11^2^ | 2.6488 | 2.6550  [0.0062] | 2.6471  [-0.0017] | 2.6693  [0.0205] | 2.6533  [0.0045] | 2.6703  [0.0215] | 2.6607  [0.0119] | 2.6724*  [0.0236] |
| ε=30 | 2.4223 | 2.4280  [0.0057] | 2.4208  [-0.0015] | 2.4412  [0.0188] | 2.4266  [0.0042] | 2.4421  [0.0197] | 2.4333  [0.0109] | 2.4441*  [0.0218] |
| **Public sector cost-effectiveness threshold = £30,000 per QALY gained** | | | | | | | | |
| ε=0 | 4.1257 | 4.1310  [0.0052] | 4.1344  [0.0087] | 4.1460  [0.0202] | 4.1372  [0.0115] | 4.1489  [0.0231] | 4.1485  [0.0228] | 4.1522*  [0.0265] |
| ε=11 | 3.2624 | 3.2694  [0.0071] | 3.2735  [0.0112] | 3.2857  [0.0233] | 3.2786  [0.0162] | 3.2873  [0.0250] | 3.2873  [0.0250] | 3.2999*  [0.0375] |
| ε=30 | 2.9902 | 2.9968  [0.0066] | 3.0006  [0.0104] | 3.0118  [0.0217] | 3.0053  [0.0151] | 3.0133  [0.0232] | 3.0133  [0.0231] | 3.0253*  [0.0351] |
| **Scenario: 20% decrease in incidence of cognitive impairment** | | | | | | | | |
| **Public sector cost-effectiveness threshold = £20,000 per QALY gained** | | | | | | | | |
| ε=0 | 3.7416 | 3.7510  [0.0095] | 3.7392  [-0.0024] | 3.7636  [0.0221] | 3.7453  [0.0037] | 3.7693*  [0.0278] | 3.7562  [0.0146] | 3.7617  [0.0201] |
| ε=11 | 2.7822 | 2.7937  [0.0115] | 2.7809  [-0.0013] | 2.8058  [0.0237] | 2.7901  [0.0080] | 2.8123*  [0.0301] | 2.7962  [0.0140] | 2.8117  [0.0295] |
| ε=30 | 2.5454 | 2.5560  [0.0106] | 2.5442  [-0.0012] | 2.5672  [0.0218] | 2.5528  [0.0074] | 2.5732*  [0.0277] | 2.5583  [0.0129] | 2.5727  [0.0273] |
| **Public sector cost-effectiveness threshold = £30,000 per QALY gained** | | | | | | | | |
| ε=0 | 4.2063 | 4.2157  [0.0094] | 4.2141  [0.0077] | 4.2302  [0.0239] | 4.2202  [0.0139] | 4.2358  [0.0295] | 4.2320  [0.0257] | 4.2380*  [0.0317] |
| ε=11 | 3.3668 | 3.3776  [0.0108] | 3.3767  [0.0099] | 3.3918  [0.0250] | 3.3854  [0.0186] | 3.3974  [0.0306] | 3.3913  [0.0245] | 3.4078*  [0.0410] |
| ε=30 | 3.0875 | 3.0975  [0.0100] | 3.0967  [0.0092] | 3.1106  [0.0232] | 3.1049  [0.0174] | 3.1159  [0.0284] | 3.1101  [0.0226] | 3.1258*  [0.0383] |
| **Abbreviation:** P: proactive; QALY: quality-adjusted life year; R: reactive; RC: recommended care; SR: self-referred; UC: usual care  ^1^ Optimal strategy marked by * and shaded in grey.  ^2^ Atkinson index elicited from the general public in England [9]. | | | | | | | | |

| **Appendix 10** Scenarios of change in intervention demand. | | | | | | | | |
| --- | --- | --- | --- | --- | --- | --- | --- | --- |
| **Scenario: 20% increase in rates of intervention demand** | | | | | | | | |
|  | **Strategy^1^** | | | | | | | |
|  | **UC** | **R** | **P** | **SR** | **R+P** | **R+SR** | **P+SR** | **RC** |
| **Public sector cost-effectiveness threshold = £20,000 per QALY gained** | | | | | | | | |
| Equally distributed equivalent per-capita societal net health benefit (EDE NHB) [incremental EDE NHB vs. UC] | | | | | | | | |
| Atkinson index ε=0 | 3.7002 | 3.7092  [0.0091] | 3.6990  [-0.0012] | 3.7266  [0.0264] | 3.7048  [0.0047] | 3.7305*  [0.0303] | 3.7165  [0.0163] | 3.7227  [0.0225] |
| ε=11^2^ | 2.7209 | 2.7329  [0.0119] | 2.7195  [-0.0015] | 2.7450  [0.0240] | 2.7312  [0.0103] | 2.7509  [0.0299] | 2.7340  [0.0131] | 2.7540*  [0.0330] |
| ε=30 | 2.4889 | 2.4999  [0.0110] | 2.4875  [-0.0014] | 2.5110  [0.0220] | 2.4984  [0.0095] | 2.5164  [0.0275] | 2.5008  [0.0119] | 2.5194*  [0.0305] |
| **Public sector cost-effectiveness threshold = £30,000 per QALY gained** | | | | | | | | |
| ε=0 | 4.1675 | 4.1768  [0.0093] | 4.1778  [0.0103] | 4.1967  [0.0292] | 4.1841  [0.0166] | 4.2010  [0.0335] | 4.1974  [0.0299] | 4.2038*  [0.0363] |
| ε=11 | 3.3190 | 3.3308  [0.0118] | 3.3307  [0.0117] | 3.3457  [0.0267] | 3.3433  [0.0243] | 3.3518  [0.0328] | 3.3465  [0.0275] | 3.3664*  [0.0474] |
| ε=30 | 3.0430 | 3.0540  [0.0110] | 3.0539  [0.0109] | 3.0676  [0.0247] | 3.0658  [0.0228] | 3.0732  [0.0303] | 3.0683  [0.0253] | 3.0872*  [0.0443] |
| **Scenario: 20% decrease in rates of intervention demand** | | | | | | | | |
| **Public sector cost-effectiveness threshold = £20,000 per QALY gained** | | | | | | | | |
| ε=0 | 3.7002 | 3.7065  [0.0064] | 3.6975  [-0.0027] | 3.7156  [0.0155] | 3.7020  [0.0018] | 3.7195*  [0.0194] | 3.7092  [0.0090] | 3.7133  [0.0131] |
| ε=11 | 2.7209 | 2.7257  [0.0048] | 2.7177  [-0.0032] | 2.7369  [0.0159] | 2.7235  [0.0026] | 2.7389  [0.0179] | 2.7274  [0.0065] | 2.7414*  [0.0205] |
| ε=30 | 2.4889 | 2.4933  [0.0044] | 2.4860  [-0.0029] | 2.5036  [0.0147] | 2.4912  [0.0023] | 2.5054  [0.0165] | 2.4948  [0.0059] | 2.5078*  [0.0189] |
| **Public sector cost-effectiveness threshold = £30,000 per QALY gained** | | | | | | | | |
| ε=0 | 4.1675 | 4.1741  [0.0066] | 4.1741  [0.0066] | 4.1847  [0.0172] | 4.1786  [0.0111] | 4.1886  [0.0212] | 4.1870  [0.0195] | 4.1912*  [0.0237] |
| ε=11 | 3.3190 | 3.3249  [0.0059] | 3.3267  [0.0077] | 3.3371  [0.0181] | 3.3326  [0.0136] | 3.3388  [0.0198] | 3.3374  [0.0184] | 3.3507*  [0.0317] |
| ε=30 | 3.0430 | 3.0484  [0.0055] | 3.0501  [0.0072] | 3.0598  [0.0169] | 3.0556  [0.0126] | 3.0612  [0.0182] | 3.0600  [0.0170] | 3.0726*  [0.0296] |
| **Abbreviation:** P: proactive; QALY: quality-adjusted life year; R: reactive; RC: recommended care; SR: self-referred; UC: usual care  ^1^ Optimal strategy marked by * and shaded in grey.  ^2^ Atkinson index elicited from the general public in England [9]. | | | | | | | | |

| **Appendix 11** Scenarios of change in GP access rate. | | | | | | | | |
| --- | --- | --- | --- | --- | --- | --- | --- | --- |
| **Scenario: 20% increase in GP access rate** | | | | | | | | |
|  | **Strategy^1^** | | | | | | | |
|  | **UC** | **R** | **P** | **SR** | **R+P** | **R+SR** | **P+SR** | **RC** |
| **Public sector cost-effectiveness threshold = £20,000 per QALY gained** | | | | | | | | |
| Equally distributed equivalent per-capita societal net health benefit (EDE NHB) [incremental EDE NHB vs. UC] | | | | | | | | |
| Atkinson index ε=0 | 3.7002 | 3.7074  [0.0072] | 3.6964  [-0.0038] | 3.7169  [0.0167] | 3.7019  [0.0018] | 3.7232*  [0.0230] | 3.7122  [0.0120] | 3.7165  [0.0163] |
| ε=11^2^ | 2.7209 | 2.7284  [0.0074] | 2.7155  [-0.0055] | 2.7336  [0.0127] | 2.7254  [0.0045] | 2.7464*  [0.0254] | 2.7297  [0.0088] | 2.7431  [0.0222] |
| ε=30 | 2.4889 | 2.4957  [0.0068] | 2.4838  [-0.0051] | 2.5005  [0.0116] | 2.4931  [0.0042] | 2.5123*  [0.0234] | 2.4969  [0.0080] | 2.5094  [0.0205] |
| **Public sector cost-effectiveness threshold = £30,000 per QALY gained** | | | | | | | | |
| ε=0 | 4.1675 | 4.1751  [0.0076] | 4.1751  [0.0077] | 4.1868  [0.0193] | 4.1809  [0.0134] | 4.1932  [0.0258] | 4.1924  [0.0250] | 4.1970*  [0.0295] |
| ε=11 | 3.3190 | 3.3274  [0.0084] | 3.3269  [0.0079] | 3.3350  [0.0160] | 3.3369  [0.0180] | 3.3471  [0.0281] | 3.3414  [0.0224] | 3.3556*  [0.0366] |
| ε=30 | 3.0430 | 3.0507  [0.0078] | 3.0503  [0.0073] | 3.0576  [0.0147] | 3.0597  [0.0168] | 3.0690  [0.0261] | 3.0636  [0.0206] | 3.0771*  [0.0341] |
| **Scenario: 20% decrease in GP access rate** | | | | | | | | |
| **Public sector cost-effectiveness threshold = £20,000 per QALY gained** | | | | | | | | |
| ε=0 | 3.7002 | 3.7085  [0.0083] | 3.6982  [-0.0020] | 3.7240  [0.0238] | 3.7033  [0.0031] | 3.7304*  [0.0302] | 3.7155  [0.0154] | 3.7202  [0.0200] |
| ε=11 | 2.7209 | 2.7299  [0.0090] | 2.7181  [-0.0029] | 2.7434  [0.0225] | 2.7215  [0.0005] | 2.7560*  [0.0351] | 2.7329  [0.0120] | 2.7462  [0.0253] |
| ε=30 | 2.4889 | 2.4972  [0.0083] | 2.4863  [-0.0026] | 2.5095  [0.0206] | 2.4893  [0.0004] | 2.5213*  [0.0323] | 2.4998  [0.0109] | 2.5122  [0.0233] |
| **Public sector cost-effectiveness threshold = £30,000 per QALY gained** | | | | | | | | |
| ε=0 | 4.1675 | 4.1758  [0.0083] | 4.1742  [0.0068] | 4.1928  [0.0253] | 4.1797  [0.0122] | 4.1996*  [0.0321] | 4.1930  [0.0255] | 4.1979  [0.0304] |
| ε=11 | 3.3190 | 3.3280  [0.0091] | 3.3267  [0.0077] | 3.3424  [0.0234] | 3.3309  [0.0119] | 3.3554  [0.0364] | 3.3418  [0.0228] | 3.3554*  [0.0364] |
| ε=30 | 3.0430 | 3.0514  [0.0084] | 3.0502  [0.0072] | 3.0645  [0.0216] | 3.0539  [0.0110] | 3.0768  [0.0338] | 3.0639  [0.0209] | 3.0769*  [0.0340] |
| **Abbreviation:** P: proactive; QALY: quality-adjusted life year; R: reactive; RC: recommended care; SR: self-referred; UC: usual care  ^1^ Optimal strategy marked by * and shaded in grey.  ^2^ Atkinson index elicited from the general public in England [9]. | | | | | | | | |

# References

1. Close J, Ellis M, Hooper R, Glucksman E, Jackson S, Swift C. Prevention of falls in the elderly trial (PROFET): a randomised controlled trial. Lancet. 1999;353(9147):93-7. doi: 10.1016/S0140-6736(98)06119-4. PubMed PMID: 10023893.

2. Shaw FE, Bond J, Richardson DA, Dawson P, Steen IN, McKeith IG, Kenny RA. Multifactorial intervention after a fall in older people with cognitive impairment and dementia presenting to the accident and emergency department: randomised controlled trial. BMJ: British medical journal. 2003;326(7380):73.

3. Spice CL, Morotti W, George S, Dent TH, Rose J, Harris S, Gordon CJ. The Winchester falls project: a randomised controlled trial of secondary prevention of falls in older people. Age and ageing. 2009;38(1):33-40.

4. Nyman SR, Ingram W, Sanders J, Thomas PW, Thomas S, Vassallo M, et al. Randomised controlled trial of the effect of Tai Chi on postural balance of people with dementia. Clinical interventions in aging. 2019;14:2017.

5. National Institute for Health and Care Excellence. Falls in older people: assessing risk and prevention. National Institute for Health and Care Excellence. 2013;Clinical Guideline 161(nice.org.uk/guidance/cg161).

6. Kwon J, Squires H, Young T. Economic model of community-based falls prevention: seeking methodological solutions in evaluating the efficiency and equity of UK guideline recommendations. BMC Geriatrics. 2023;23.

7. Kwon J, Lee Y, Young T, Squires H, Harris J. Qualitative research to inform economic modelling: a case study in older people’s views on implementing the NICE falls prevention guideline. BMC Health Services Research. 2021;21(1):1-19.

8. Iliffe S, Kendrick D, Morris R, Masud T, Gage H, Skelton D, et al. Multicentre cluster randomised trial comparing a community group exercise programme and home-based exercise with usual care for people aged 65 years and over in primary care. Health technology assessment (Winchester, England). 2014;18(49):vii-105. doi: <https://dx.doi.org/10.3310/hta18490>.

9. Asaria M, Griffin S, Cookson R. Distributional Cost-Effectiveness Analysis: A Tutorial. Medical decision making. 2016;36(1):8-19. doi: 10.1177/0272989X15583266.
